# Effects of acupuncture on patients with Alzheimer's disease: Protocol for a systematic review and meta-analysis

**DOI:** 10.1097/MD.0000000000014242

**Published:** 2019-01-25

**Authors:** Shujun Shao, Yinshan Tang, Yu Guo, Zhaoyang Tian, Dulian Xiang, Jihong Wu

**Affiliations:** a School of Acupuncture-Moxibustion and Tuina, Beijing University of Chinese Medicine, Beijing; b Department of Rehabilitation in Traditional Chinese Medicine, The Second Affiliated Hospital of Zhejiang University School of Medicine, Hangzhou; c Department of Traditional and Chinese Medicine, Darenhe Clinic affiliated to Beijing Ciaijia Care service Co., Ltd, Beijing, China.

**Keywords:** acupuncture, Alzheimer's disease, protocol, systematic review

## Abstract

**Background:**

Alzheimer's disease (AD) is the leading progressive neurodegenerative disease worldwide, accompanied with nonreversible cognitive impairments. Acupuncture, as the traditional Chinese technique, is widely applied in clinical trials for AD. The aim of this review is to evaluate the efficacy and safety of acupuncture in the aspect of antidementia.

**Methods:**

Randomized controlled trials (RCTs) related to acupuncture treatment targeting AD will be collected. We will search the following 4 databases of electronic English resources, including PubMed, Embase, MEDLINE, Web of Science, and 4 Chinese databases, namely CNKI, CBM, VIP, and Wanfang database. All the RCTs will be searched from their inception to November 2018. After screening the studies, a meta-analysis of RCTs will be carried out. Subsequently, the assessment of bias risk, data synthesis, subgroup analysis will be conducted using RevMan V.5.3.5 software if the setting condition is met.

**Results:**

This systematic view and meta-analysis will assess the efficacy and safety of acupuncture intervention on AD patients, which is fundamentally based on current published evidence, and provide a high-quality synthesis for clinical practitioners of treating AD with acupuncture,

**Conclusion:**

The summary of our systematic view will determine whether acupuncture intervention could be an efficient and feasible approach to the treatment of AD patients.

## 1 Introduction

Alzheimer's disease (AD) is the leading prevalent form of dementia in elderly globally with an increasing tendency.^[1,2]^ Pathological symptoms of AD advances from mild to moderate to severe differs in person. However, cognitive function generally deteriorates as the disease progresses.^[3]^ At initial stage, subtle cognitive decline that is well viewed as precursor symptom to AD will be observed,^[4]^ While as the neuronal damage proceeds, moderate cognitive decline will occur, such as memory loss and spatial disorientation, especially forgettable of recent events, accompanied by obvious motor dysfunction like dysphagia.^[5]^ During this period, patients become more subject to mood swings, and can be easily irritated. In latter stage, people lose the ability of remembering easy things, such as dress up, unable to communicate and become bed-bound, subsequently full-time care should be undertaken. Eventually, AD-related pneumonia may lead to the death of AD patients.^[6]^

Accordingly, AD not only adversely affects physical functions, but also negatively affects the quality of life, resulting in extensively increasing financial burden on AD-related medical care system. As reported, there were over 47 million people worldwide suffering from dementia, and the figure is estimated to increase to 131.5 million by 2050.^[7]^ Currently, the prevalence of AD is increasing globally, especially in developing countries.^[8]^ The worldwide costs of dementia were estimated to be proximately United States (US) $818 billion in 2015, which has increased 35% in the last 5 years.^[9]^ In China, the total annual socioeconomic costs on long-term care and hospital service were around US$167.74 billion in 2015.^[10]^ The expenditures in America were expected to be around $226 billion that spent on patients with dementia in the year 2015.^[6]^ Considering the prevalence and societal impacts, addressing AD should be currently undertaken as a policy priority.

The typical pathological features in the brains of AD patients are amyloid-β (Aβ) senile plaques accumulation and neurofibrillary tangles, which ultimately lead to atrophy and destruction of neurons that result in synapse loss and cognitive impairments.^[2]^ The deposition of Aβ is proved to be involved in neuron to neuron communication procedures at synapses and thus accelerate cell death. Meanwhile, the abnormal phosphorylation of tau also contribute significantly to the pathogenesis of AD. Converging studies revealed that Aβ and tau exert neurotoxic effects separately and also synergistically.^[1,11,12]^ However, most therapeutic intervention available in the clinic are targeting at Aβ or tau separately, hence the treatments effects are not feasible to fully reverse the disorder. Thus, AD maybe a collection of distinct diseases that adequate strategies should be adopted separately to hinder the progression of Alzheimer's pathogenesis.

Pharmacological treatment available to AD mainly working on anti-AChE and via inhibiting Aβ-induced neurotoxity,^[13,14]^ which has been reported to be with gastrointestinal consequence,^[14]^ and possibly reflect the skepticism in effectiveness of drugs intervention. Conservative management in approach to AD such as psychological consultation are considered to delay the progression of AD. However, no curative response is achieved. Because of the above-mentioned limitations and uncertainty of how to better alleviate and prevent AD with fewer adverse effects in clinic trials, new effective and affordable therapy are urgently needed in antidementia health sector.

Acupuncture is based on the theory of meridians and acupoints that plays an essential role in regulating and maintaining the innate *yin* and *yang* in bodily system. The application of acupuncture is performed on a holistic perspective, since defensive Qi would be strengthened to defeat exogenous factors. It has been confirmed that acupuncture could protect neurons from deterioration and promote axonal regrowth on neurodegenerative diseases, such as AD.^[15]^ Moreover, accumulative lines of evidence have demonstrated that acupuncture could exert apparently regulatory effects on glucose metabolism intake rate.^[16,17]^ as glucose metabolism is enhanced to compensate neuronal dysfunction while neurons are presented with brief hyperglycolytic state. Recently, acupuncture is widely applied by clinicians for treating AD as a disease-modifying therapy,^[18,19]^ while few associated side effects has been reported. Although abundant clinical researches exist about acupuncture intervention to AD, a systematic evaluation and meta-analysis about it's efficacy and safety still remains methodologically insufficient. The aim of this study is to analyze various RCTs, further summarize and critically evaluate the comparative effectiveness and safety of acupuncture intervention in clinical trials, attempting to aid the field to advance medical treatment to AD.

## 2 Methods

This protocol of systematic review has been registered on the international prospective register of systematic review (PROSPERO), the registration number is CRD42018117845. The protocol will be strictly developed under the guidelines of Preferred Reporting Items for Systematic Reviews and Meta-Analyses protocols (PRISMA-P).^[20]^

### 2.1 Eligible criteria for study selection

#### 2.1.1 Types of studies

Only the RCTs of acupuncture treating AD will be included in this study, without publication or language restriction. While non-RCTs will be ruled out, including studies such as case report, and the study without sufficient information about the randomized method or process.

#### 2.1.2 Types of participants

Participants suffering from AD will be included, regardless of the restriction of age, gender, nationality, and education background. However, participants with dementia but are diagnosed to be vascular dementia (VD) or mixed dementia (MD) will be excluded.

#### 2.1.3 Types of interventions

##### 2.1.3.1 Experimental interventions

We will include acupuncture and acupuncture related therapies (regular acupuncture, scalp acupuncture, auricular acupuncture, electroacupuncture, fire needling, intradermal needling, and catgut embedding acupuncture) to comprehensively describe the effects of acupuncture on AD patients. Clinical trials with other forms of stimulation including moxibustion, bleeding therapy, cupping, laser acupuncture, pharmacoacupuncture, acupotomy, point injection, or acupressure will be excluded. Additionally, limitation to intervention intensity, frequency and duration will not be involved.

##### 2.1.3.2 Control interventions

Studies that are interfered with no treatment, an acupuncture-like intervention or with conventional antidementia drugs will be considered as control groups. Additionally, studies compare acupuncture plus the concomitant of other treatment with that treatment will also meet the control group including criteria. However, studies comparing acupuncture with other TCM treatments will be ruled out.

#### 2.1.4 Types of outcome measures

##### 2.1.4.1 Primary outcomes

The brain biomarker imaging examinations such as positron emission tomography (PET), magnetic resonance imaging (MRI) will be performed to identify the pathological changes as the primary outcomes. Also, the improvement of memory and learning ability, spatial disorientation, motor function and better emotional management of depression or anxiety will be assessed. Relative validated scales such as MMSE (Minimum Mental State Examination), ADL (activity of daily living), NPI (neuropsychiatric inventory), etc., will be employed to evaluate the physiological and psychiatrical changes.

##### 2.1.4.2 Secondary outcomes

The following subjects including evaluation of quality of life (QOL) performed by QOL-AD scale, as well as the side effects and complications caused by the intervention of acupuncture for AD will be assessed as the secondary outcomes.

### 2.2 Search methods for the identification of studies

#### 2.2.1 Electronic searches

We will screen a comprehensive literature from the following databases: 4 English databases including PubMed, Excerpta Medica Database (Embase), Medline, Web of Science and 4 Chinese databases involving China National Knowledge Infrastructure (CNKI), Chinese Biomedical Literature Database (CBM), Chinese Scientific Journal Database (VIP) and Wanfang database, without limitations of publications and countries. The retrieved trials will be performed on databases from the inception to November, 2018, unpublished literature will also be searched as supplement. The following search items will be adopted: AD, Alzheimer, Alzheimer's disease, dementia, cognitive impairments, neurodegeneration, acupuncture, acupuncture treatment, acupunctue needling, scalp acupuncture, auricular acupuncture, ear acupuncture, electroacupuncture, fire needling, intradermal needling, catgut embedding, and embedding acupuncture. To ensure the same searching terms in both Chinese and English database, an equivalent translation of the search terms will be adopted. Here, the search strategy for PubMed is presented in Table 1 and we will make relative modifications in accordance to the requirements.

**Table 1:**
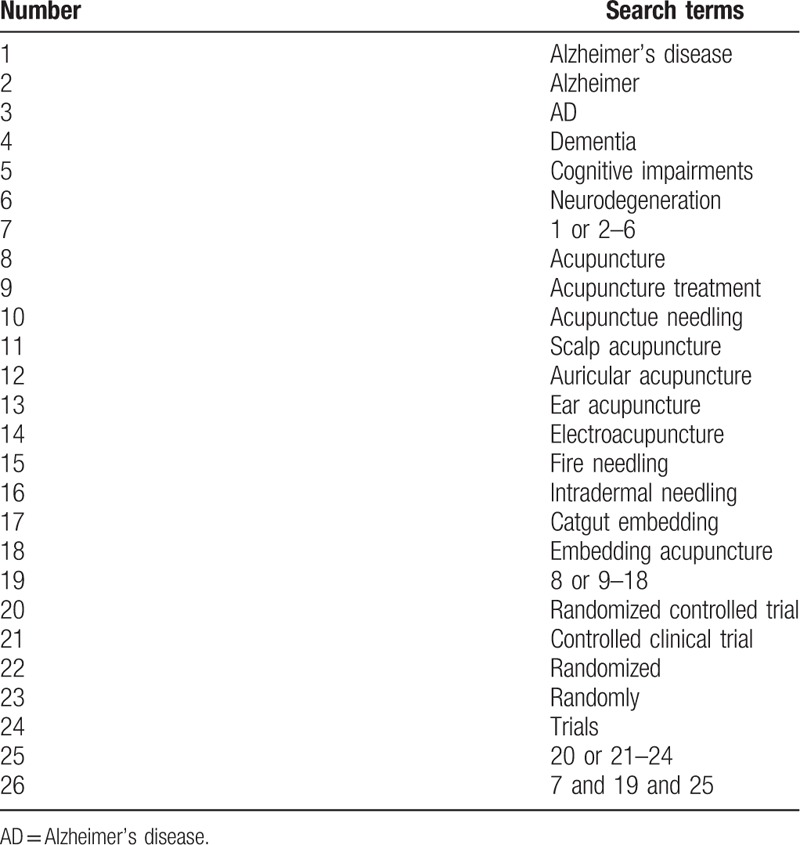
Search strategy for PubMed.

#### 2.2.2 Searching other resources

Additionally, the following Chinese medical journals will also be searched as complement: Chinese Acupuncture and Moxibustion (1985–2018), Acupuncture Research (1985–2018), China Journal of Traditional Chinese Medicine and pharmacy (1985–2018). Meanwhile, relevant conference papers will be manually retrieved. At the same time, we will also search the WHO International Clinical Trials Registry Platform (ICTRP) to contain new trails pertaining to the theme.

### 2.3 Data collection and analysis

#### 2.3.1 Study selection

**Figure 1:**
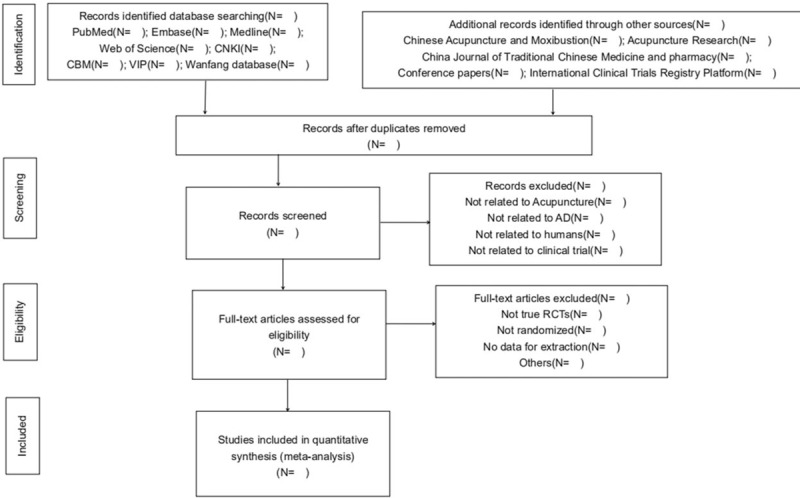
Preferred Reporting Items for Systematic Reviews and Meta-Analysis (PRISMA) flow diagram.

To ensure that all reviewers get a comprehensive understanding of the purpose and process of this study, we will organize a group meeting to delivery relative information before the conduction of this study. Two authors (SS and YT) will screen the titles and abstracts respectively to extract potential eligible articles, results that are duplicated will be excluded. Further identification will be subsequently carried out by reviewing the full-text and analysis consideration to select eligible studies. Then, all reviewers will have a group discussion on the consistency of all the studies included, exclude and eliminate those are not up to the theme topic till final team consensus arrived. The selection process is fully elucidated in the following PRISMA flow diagram (Fig. 1).

#### 2.3.2 Data extraction and management

An electronic form will be established to extract substantial contents, and then filled by Shujun Shao and Yinshan Tang independently, which is consisted of the first and corresponding author, inception time, trial designate, characteristics of participants, interventions, duration, outcomes, adverse effects, and other specific information. Disagreements will be solved by group discussion or consult seniors. However, if we fail to reach the consensus, the authors of trials will be contacted for further details and verification.

#### 2.3.3 Assessment of bias risk and quality of included studies

Based on the guidelines of Cochrane collaboration's tool, the assessment of methodological quality of included study will be respectively executed by Shujun Shao and Yinshan Tang to evaluate the potential bias risk. Six perspectives pertained to bias risk including generation of random sequence, allocation concealment, blinding of outcome assessors, incomplete of outcome data, selective outcome reporting, and other issues will be evaluated. The classification of bias risk is graded as low, high, and unclear bias.

#### 2.3.4 Measurements of treatment effect

For continuous data, the mean difference (MD) will be performed to evaluate the treatment effect, under which the same method or measure scale are adopted. As for enumeration data, the relative risk (RR) will be performed to estimate the treatment effect. The standardized MD with 95% confidence interval (CI) or RR with 95% CI will be employed to indicate the effect size.

#### 2.3.5 Managing missing data

We will primarily consider the possible reasons involved if any data information is not sufficient in included trials. If possible, the 2 viewers (SS and YT) will contact the corresponding author by email or phone, requesting for adequate information and details of the studies included. However, if the author is not available or sufficient information cannot be obtained, we will have a group discussion and analysis based on the current information. Meanwhile, the potential impact of missing data will be taken into account and relative discussion will be presented in the result section.

#### 2.3.6 Assessment of heterogeneity

Based on the guidelines in Cochrane Handbook for systematic review, we will perform a standardized chi-squared test (α = 0.1) and *I*^2^ value, respectively, to assess the heterogeneity. If *I*^2^ ≤ 50%, the studies included will indicate no presence of meaningful heterogeneity, and then a fixed-effect model will be employed to evaluate the effect sizes. While, if *I*^2^ > 50%, indicating the heterogeneity among studies are statistical significant, and a random-effect model will be adopted.

#### 2.3.7 Assessment of reporting biases

When the number of trials included in the meta-analysis is more than 9, a visual asymmetry on the funnel plot will be developed to evaluate the existing bias of included studies.

#### 2.3.8 Data synthesis

The synthesis will be carried out using RevMan V.5.3.5 provided by Cochrane Collaboration. In accordance to Cochrane guideline, if *I*^2^ < 50%, a fix-effect model will be employed to evaluate MD and RR. Otherwise, the source of heterogeneity will be analyzed using a random-effect model to exclude obvious clinical heterogeneity. If evident heterogeneity is found between studies, we will conduct a subgroup analysis to explore the possible reasons attributing to this statistical heterogeneity, and present a reasonable explanation.

#### 2.3.9 Subgroup analysis

To detect the heterogeneity between groups under the condition that the existing studies are sufficient, we will conduct a subgroup analysis to explore the feasibility of the review conclusions. The subgroup analysis will be carried out to interpret the robustness of studies according to following aspects:

1. Gender and age of patients.
2. Different forms of acupuncture intervention (needles, frequency, tensity, points, duration, and treatment session).
3. the severity of AD.

#### 2.3.10 Sensitivity analysis

After removing the low-quality studies, we will perform a sensitivity analysis to evaluate the robustness of the results according to following aspects: sample size, missing data, and methodologically quality. The sensitivity analysis is conducted to exclude the inappropriate trials without random generation.

#### 2.3.11 Grading the quality of evidence

The Grading of Recommendations Assessment, Development and Evaluation (GRADE) guidelines will be employed to evaluate the quality of evidence for primary outcomes. The strength of evidence will be graded it into very low, low, moderate or high level.

#### 2.3.12 Dissemination and ethics

Formal ethical approval is not required in this protocol. We will collect and analyse data based on published studies, and since there is no patients involved in this study, individual privacy will not be under concerns. The results of this review will be disseminated to peer-reviewed journals or submit to related conferences.

## 3 Discussion

Alzheimer's disease is a deleterious neurodegenerative disorder, characterized with a complex multifactorial pathology. The clinical progression of AD is manifested with obvious defining decline in cognitive abilities, and various motor dysfunction occur throughout the disease spectrum.^[2,3,5]^ Currently, socioeconomic costs on AD long-term attendance and hospital care are increasing annually, laying burgeoning obstacles on social development.^[6,8]^ Unfortunately, there exists no comprehensive approaches to prevent and ameliorate AD-like dementia.

Acupuncture, originated from ancient China, has been widely applied in clinic for a long time. According to Traditional Chinese Medicine (TCM) theory, needles inserting into specific documented acupoints can generally dredge meridians and relieve blockage in the flow of Qi and blood, which is considered to be the fundamental energy activate and maintain the holistic body system. Also, acupuncture can strengthen congenital and required Qi to promote health, thereby create a therapeutic effect on bodily organs and functions, defending body from invasive factors attack. Additionally, as a treatment to AD, acupuncture intervention primarily involves inserting and stimulating certain points has been identified to modulate neuron synaptic plasticity, resulting in mitigation to cognitive impairments.^[18,19]^ In recent years, acupuncture for treating AD has gained increasing popularity across the globe, further reinforce the possible efficacy and safety of acupuncture treatment to AD.

The present review is conducted to assess the effectiveness and safety of acupuncture in treating AD on evidence-based literature, both RCTs from home and abroad will be extracted, aiming to establish a more convincing preference for clinicians and investigators when treating AD with acupuncture.

However, albeit every effort we made to minimize the errors and deviations, it should also be noted that the several restrictions involved regarding our systematic review. First, the inclusion of studies solely based on the usage language of Chinese and English, and the papers written in other languages such as Korean, Japanese, or Spanish will be excluded, which may lead to uneven distribution of patients. Second, various types of acupuncture intervention, frequency, duration, gender and age of patients, severity of AD all may contribute to the heterogeneity arise. Awareness of the potential factors that may cause heterogeneity, we will appropriately plan and power our study to reduce bias.

## Author contributions

SS and YT conceived the idea of developing the research. SS, YT and Jihong Wu contributed to the design of search strategy. The manuscript was drafted by SS and YT. SS and YT will independently collect and extract eligible studies. YG, ZT, and DX will assess the bias risk and deal with missing data. JW is the arbitrator of this study, and guarantees the proper development of this review. All the authors participated in this study critically revised the final version of manuscript, and confirmed on the publication of this protocol.

Shujun Shao orcid: 0000-0003-2776-8215.
